# Enteric methane emissions, rumen fermentation, and milk composition of dairy cows fed 3-nitrooxypropanol and *L*-malate supplements

**DOI:** 10.3389/fvets.2024.1479535

**Published:** 2024-12-20

**Authors:** Xiaokang Zhou, Shuaiqi Fu, Gaiying Li, Zhaohui Yao, Xingjie Du, Yan Zhang, Tengyun Gao

**Affiliations:** College of Animal Science and Technology, Henan International Joint Laboratory of Nutrition Regulation and Ecological Raising of Domestic Animal, Henan Agricultural University, Zhengzhou, Henan, China

**Keywords:** methane emissions, 3-nitrooxypropanol, 3-nitrooxypropanol plus *L*-malate, milk composition, milk fat

## Abstract

Twenty-four cows were used in a randomized complete block design. Cows were assigned to three groups: (1) Control, (2) 3-nitrooxypropanol (NOP) of 200 mg/kg feed dry matter (10% NOP), and (3) NOP × MAL (10% NOP at 200 mg/kg feed dry matter plus 99% *L*-malate at 10 g/kg feed dry matter). Cows were fed for 10-wk. NOP did not affect dry matter intake (DMI) or milk yield, whereas NOP × MAL decreased DMI but did not affect milk yield. Average methane production decreased by 54% in NOP and by 51% in NOP × MAL. Both NOP and NOP × MAL increased concentrations of milk fat and protein. In addition, concentrations of short-chain fatty acids and total saturated fatty acids increased in both NOP and NOP × MAL. However, total monounsaturated fatty acids and total polyunsaturated fatty acids only increased in NOP × MAL.

## Graphical abstract

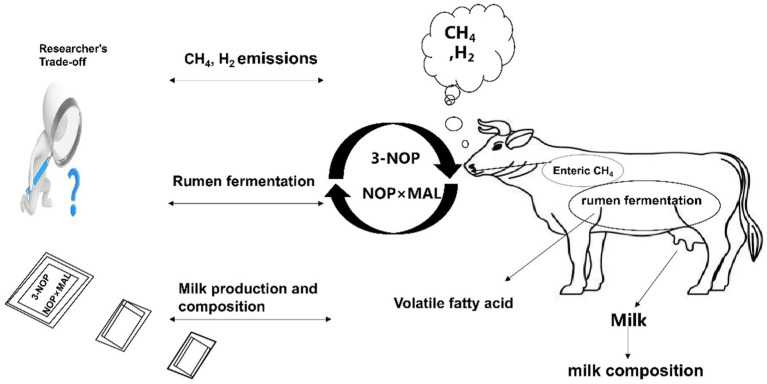

## Highlights

- Average methane production decreased by 54% in NOP and by 51% in NOP × MAL.
- Both NOP and NOP × MAL decreased the molar ratio of acetate-to-propionate.
- Both NOP and NOP × MAL increased concentrations of milk fat and protein.

## 1 Introduction

Methane (CH_4_) produced in ruminant intestines is a greenhouse gas with warming potential. Over 100 years, the global warming potential of CH_4_ is 28–34 times that of carbon dioxide (1), but its greenhouse effect is 80 times that of carbon dioxide in the 10–20 years after its release (2). Methane in rumen is mainly produced by several methanogenic archaea that reduce carbon dioxide (CO_2_) through hydrogen gas (H_2_), and it is chemically very stable (3, 4). Methane is ultimately excreted in the form of rumen fermentation by-products (5). Globally, intestinal CH_4_ emissions of ruminants account for approximately 3–5% of total greenhouse gas emissions (6) and 2–12% of total ruminant dietary energy intake (7). Reductions in CH_4_ emissions from the intestines of ruminants can be a means to achieve the goal of the Paris Agreement, which aimed to stabilize the global climate– at 1.5°C to 2°C above the preindustrial level (8). Therefore, it is urgent to reduce CH_4_ emissions and to develop strategies to increase the energy utilization rate of ruminant diets. Meanwhile, to address climate change at the national level, the Chinese government has set the strategic goals of achieving peak carbon dioxide emissions in 2030 and carbon neutrality in 2060.

In exploring how to reduce intestinal CH_4_ emissions, ruminant diets have been supplemented with tannins, saponins, monensin, bromochloromethane, and vegetable oil (9–11). Although those dietary additives reduced enteric CH_4_ emissions to a certain extent, they also likely decreased the digestibility and production performance of animals and had some toxic effects on animals with unsustainable intestinal CH_4_ emissions (10–14). Therefore, there are limitations with dietary supplements in animal production.

Feeding a supplement to ruminants with at least one organic molecule substituted by at least one nitrooxy group at any position has recently been shown to be very effective in reducing CH_4_ production with no negative effects on rumen fermentation (15). However, when the nitrooxy group is replaced by other chemical groups with similar physical and chemical properties, the inhibitory effect on CH_4_ production is lost. Thus, as reported by the patent inventors (15), the nitrooxy group is the key to reducing CH_4_ emissions. Among several organic compounds listed by the inventors that are substituted by at least one nitrooxy group at any position, 3-nitrooxypropanol (NOP) has been the most studied. The nitrooxy group in NOP specifically binds to coenzyme M reductase (MCR), and as a result, the nickel ion in its nickel enzyme is oxidized from +1 to +2 to inactivate MCR, which further continuously inhibits CH_4_ emissions (16). Although the results of NOP studies are slightly different, it consistently and continuously reduces CH_4_ emissions and increases hydrogen gas (H_2_) emissions (17–20). Hydrogen gas is a high-energy gas, and when hydrogen produced by ruminant fermentation in a diet cannot be effectively used by animals, it is an indirect waste of diets (21, 22). Therefore, a reasonable approach is urgently needed to promote H_2_ use by ruminants.

In the process of biological oxidation in animals, *L*-malic acid is used as a hydrogen or an electron transporter to transfer hydrogen to mitochondria in rumen microbes and the mitochondria in the cow for oxidation to generate energy (23–25). Milk yields increase when *L*-malic acid is fed to dairy cows or dairy goats (26–28). The increases are likely because hydrogen is transferred to cell mitochondria by *L*-malic acid, and then, H^+^ is oxidized into extra energy to improve animal production performance.

NOP is listed by Duval and Kindermann (15) and therefore, it was selected as the inhibitor of CH_4_ emissions in one treatment group in the experiment in this study to determine whether the effect on reducing CH_4_ emissions has a similar conclusion to that of previous researchers who studied NOP. NOP plus *L*-malic acid (NOP × MAL) was used in the other treatment group to determine whether H^+^ was oxidized to adenosine triphosphate (ATP) by cell mitochondria to provide energy. Therefore, the purpose of this study was to determine the effects of NOP and NOP × MAL supplementation on CH_4_ emissions, milk yield, rumen fermentation, and milk composition of dairy cows in the middle lactation period.

## 2 Materials and methods

All animals involved in the experiment were cared for according to the guidelines of the Animal Care and Use Committee of Henan Agricultural University (Zhengzhou, China). All experimental procedures were reviewed and approved by the committee. The NOP (10% NOP) compound was developed by DSM Nutritional Products Ltd. (Kaiseraugst, Switzerland) and was applied at 200 mg/kg feed dry matter. The organic acid MAL (99% MAL) was developed by Changmao Biochemical Engineering Institution (Changzhou, China) and was applied at 10 g/kg feed dry matter. The NOP dosage were supplemented in the diet of dairy cows in the middle lactation period according to Duval and Kindermann (15) and the report on malic acid used by previous researchers in dairy cows (29, 30). The milk of the cows in this experiment was abandoned for 7 days, and the cows were still used for production.

### 2.1 Experimental design, diet, and treatment

Twenty-four Holstein cows (parity 3) with similar age, weight (659 ± 20 kg), lactation stage (115 ± 10 d), and milk yield (24.6 ± 1.6 kg/d) were used in a 10-wk randomized complete block design and it consisted of 7-d sample collection. All cows were placed in a shaded open barn.

All cows were milked twice daily at 0600 and 1800 and were fed with a total mixed ration (TMR) (Table 1) diet twice daily at 0700 and 1900. During the whole experiment, cows could freely intake diet and drinking water. Control (CON) animals were not fed either supplement. The two treatment groups included animals fed 10% NOP at 200 mg/kg feed dry matter or 10% NOP at 200 mg/kg feed dry matter plus 99% MAL at 10 g/kg feed dry matter. The NOP and MAL were added as powders to the TMR and premixed, which allowed the dairy cows to consume the supplements all day by consuming diet.

**Table 1:** Ingredients and nutritional composition of the experimental diet.

Item, % dry matter (unless otherwise noted)	Total
Ingredient	
Dry ground corn	28.5
Soybean meal	8.4
Corn silage	19.3
Alfalfa haylage	17.8
Soyhull	8.1
Oat hay	7.5
Cottonseed, whole	5.0
CaHPO_4_	0.5
Salt	0.5
CaCO_3_	0.9
Molasses	3.0
Mineral and vitamin premix^1^	0.5
Nutrient composition	
DM^2^, %	47.0
CP^3^	16.5
NDF^4^	29.9
ADF^5^	17.9
Ether extracts	3.2
Ash	9.0
Ca	1.0
P	0.4

1 Premix per 1 kg of diet: vitamin A, 1,500 KIU/kg; vitamin D_3_, 350 KIU/kg; vitamin E, 8,000 IU/kg; niacin, 5,000 mg/kg; biotin, 200 mg/kg; β-carotene, 600 mg/kg; Mn, 3,500 mg/kg; Cu, 2,500 mg/kg; Zn, 12,500 mg/kg; iodine, 200 mg/kg; Co, 60 mg/kg; Se, 65 mg/kg.

2 DM, dry matter.

3 CP, crude protein.

4 ADF, acid detergent fiber.

5 NDF, neutral detergent fiber.

### 2.2 Data and sample collection

When animals were fed, the ration provided to cows and the portion of diet rejected by all cows were recorded. Recording daily feeding and refusal allowed the amount of TMR fed to cows to be adjusted based on a daily refusal of 10%. Because the contents of NOP and MAL supplements were consumed by dairy cows after premixing with the TMR, they were not determined in the discarded diet. To determine the chemical composition of the diet, a TMR diet sample (approximately 600 g) was collected on days 69 and 70. Samples of TMR were dried in a forced-ventilation drying oven at 65°C for 72 h and then ground to pass through a 1-mm sieve. Samples were stored at 4°C until chemical analysis of feed components. The TMR diet was adjusted every 2 weeks to ensure that the concentrate-to-roughage ratio (5:5) fed to all animals was similar. The weights of cows were measured at the beginning and the end of this experiment. In addition, milk yield was continuously recorded from 10-wk. Milk samples collected in the morning and evening were mixed and divided into two 50-mL sterile test tubes. One milk sample was mixed with 2-bromo-2-nitropropane-1,3-diol and stored at 4°C, and the other sample was stored at −20°C until analysis of milk components and fatty acids (FAs). In addition, to determine enzyme activities in the serum of dairy cows, blood was collected from the cow tail vein with disposable venous blood collection needles and negative pressure blood collection tubes. Blood was collected before cows were fed at 0700 on days 69 and 70. Blood was centrifuged at 2,000 × *g* for 20 min at 4°C, and then, the serum was removed with plastic straws and stored at −30°C until analysis of enzyme activity.

Rumen fluid was collected before feeding at 0700 on days 69 and 70. Immediately after collection, and a 10-mL sterile syringe was used to inject rumen fluid into a 10-mL frozen tube, which was quickly stored at −80°C in liquid nitrogen until analysis of rumen microorganisms. In addition, the rumen fluids were filtered through two layers of filter gauze. Filtered samples were put into 50-mL centrifuge tubes and centrifuged at 4,000 × *g* for 20 min at 4°C, and then the samples were immediately stored at −20°C until analysis of volatile fatty acids (VFAs) and N-NH_3_.

Methane emissions from the guts of all dairy cows were measured by using sulfur hexafluoride (SF_6_) tracer gas technology (31) for five consecutive days (days 66–70). Halters and polyvinyl chloride neck yokes (internal capacity of approximately 2 L) modified by Johnson et al. were used as the devices to collect CH_4_ gas. In addition, the design of the halters and yokes could be allowed to a half of 100% reduction in yoke vacuum pressure through the connected stainless steel capillary tube over 24 h. Pure SF_6_ brass permeation tubes were made by members of our laboratory and were stored in an anaerobic environment at 39°C for 3 months before the experiment began to determine the permeation rates. For example, the average release rates (mean ± SD) of the groups 1, 2 and 3 were 5.16 ± 0.33, 4.91 ± 0.38, and 5.84 ± 0.36 mg/d, respectively. One week before gas collection, the SF_6_ permeation tubes with known permeation rates were put into the rumen through a rumen catheter. In the first week of collection, the halters, yokes, and stainless steel capillary tubes were worn by cows to measure CH_4_ emissions.

At the beginning of measuring CH_4_ emissions (days 66–70), the air in the yokes was pumped out at 0600 daily to induce negative pressure, followed by placing the yokes on the cows. Halters and cow yokes were replaced every 24 h. High-purity nitrogen (N_2_) was used daily to check whether there was pipeline blockage in the halters. To obtain a representative sample, the yokes were pressurized with N_2_ to induce positive pressure. Three 100-mL subsamples were collected from each yoke using syringes and then injected into three corresponding 100-mL gas sampling bags (Dalian, China), which were used to analyze background concentrations of CH_4_, SF_6_, and H_2_. At the end of each sampling, the yokes were pressurized with N_2_ three times and then decompressed. They were pressurized again and then remained under pressure until the next day to check whether there were any leaks. If there were no leakages, they were used again to collect samples. To calculate average daily CH_4_ emissions, background yokes were treated in the same way as cow treatment yokes.

### 2.3 Sample analyses

Dried TMR samples were ground by a pulverizer and passed through a 1-mm mesh screen and then sent to the feed and detection analysis laboratory of Henan Agricultural University to determine the dry matter (DM), crude protein (CP), acid detergent fiber (ADF), and ash by AOAC International official methods 930.15, 990.03, 973.18, and 942.05, respectively (32). Crude fat was determined by AOAC methods 2003.05 (33). The neutral detergent fiber (NDF) was determined by the method of Van Soest et al. (34). Concentrations of phosphorus (P) and calcium (Ca) were determined according to the guidelines for (35). Concentrations of milk fat, protein (CP), lactose, and milk urea nitrogen (MUN) were measured using infrared spectroscopy with a Milk Composition Somatic Cell Analyzer (CombiFossTM-7; Beijing, China) at the Henan Dairy Production Performance Testing Institution (Zhengzhou, China). Concentrations of FAs were measured using a gas chromatograph (GC-2010 Plus; Shanghai, China) at the Qingdao Yixin Testing Technology Service Institution (Qingdao, China). Residues and metabolites of NOP in milk were measured by using high-performance liquid chromatography (U 3000; Shanghai, China). After collection, the pH of rumen fluid was immediately measured with a pH meter (ST-20; Shanghai, China). To determine the concentration of VFAs in rumen fluid, rumen fluid samples were centrifuged at 10,000 × *g* for 20 min, and then, supernatants were analyzed by gas chromatography according to the method described by Schlau et al. (61). The concentration of N-NH_3_ in rumen fluid was determined according to the guidelines described by Ivan et al. (62).

Rumen contents stored at −80°C were sent to Shanghai Meiji Biomedical Technology Institution (Shanghai, China) on dry ice. Frozen rumen content (approximately 2 g) was thawed on ice, and total DNA was extracted by a bead-beating method to determine the copy numbers of total bacteria, methanogenic archaea, and protozoa (36). Following DNA extraction, total populations of bacteria and methanogenic archaea were measured and analyzed by qPCR using the primer pairs U2 (37) and uniMet (38), respectively, and total protozoa copy numbers were measured and analyzed by qPCR and SYBR-green chemistry with the primer pair P-SSU-316F (39) and P-SSU-539R (40). Serum of the three treatment groups were sent to Nanjing Jiancheng Institute of Bioengineering (Nanjing, China), and activities of the enzymes for the malate dehydrogenase (MDH), phosphoenolpyruvate carboxykinase (PCK), pyruvate kinase (PK), and citrate synthase (CS) were determined spectrophotometrically by using enzyme-linked immunosorbent assay (ELISA) detection kits (Nanjing, China) (41).

In the gas detection center of Henan Agricultural University, gas chromatography was used to detect the background concentration of CH_4_ in the gas obtained from cow yokes by hydrogen flame ionization (GC1120; Shanghai, China) and also by detecting the background concentration of SF_6_ by electron capture detection (GC-4000A; Beijing, China). Methods were according to those described by Johnson et al. (42). The background concentration of H_2_ was measured by a pump-type H_2_ detector (SKY 2000; Beijing, China). In addition, the treatment of background yokes was same to that of cow yokes. However, the background concentration of SF_6_ was usually very small compared with that of cow yokes, and thus, it was ignored. In the calculation of CH_4_ emissions, the representative samples and data for 3 d in whole period were selected according to Hristov et al. (63). The background CH_4_ level was only subtracted from the CH_4_ concentration in the yokes of dairy cows, according to Johnson et al. (42). To facilitate statistical analysis, daily CH_4_ emissions were averaged for all cows.

### 2.4 Calculations and statistical analyses

The methane emission rate (QCH_4_) was calculated from the measured concentrations of CH_4_ ([CH_4_]_y_) and SF_6_ in the yokes, the CH_4_ ([CH_4_]_b_ concentration in the background yokes, and the known release rate of SF_6_ (QSF_6_) (42) as follows:

${QCH}_{4}={QSF}_{6}\times \left({\left[{CH}_{4}\right]}_{y}-{\left[{CH}_{4}\right]}_{b}\right)/\left[{SF}_{6}\right]$.

All data were analyzed as the analysis of variance model by using the one-way ANOVA program in SPSS 19.0 (2010; SPSS Inc., Chicago, IL, USA). The effects of NOP and the interaction of NOP × MAL on CH_4_, DMI, milk yield, milk composition, VFAs, total bacteria, total methanogenic archaea, and total protozoa were analyzed. When there was a significant difference, multiple comparisons were made to determine the differences among the three treatments by using the Duncan. Differences were considered significant at *p* ≤ 0.05, and significant trends were recognized at 0.05 < *p* ≤ 0.10.

## 3 Results and discussion

### 3.1 Effects of NOP and NOP × MAL on dry matter intake, methane emissions, milk yield, feed efficiency, milk composition, and serum enzyme activity of dairy cows

**Figure 1:**
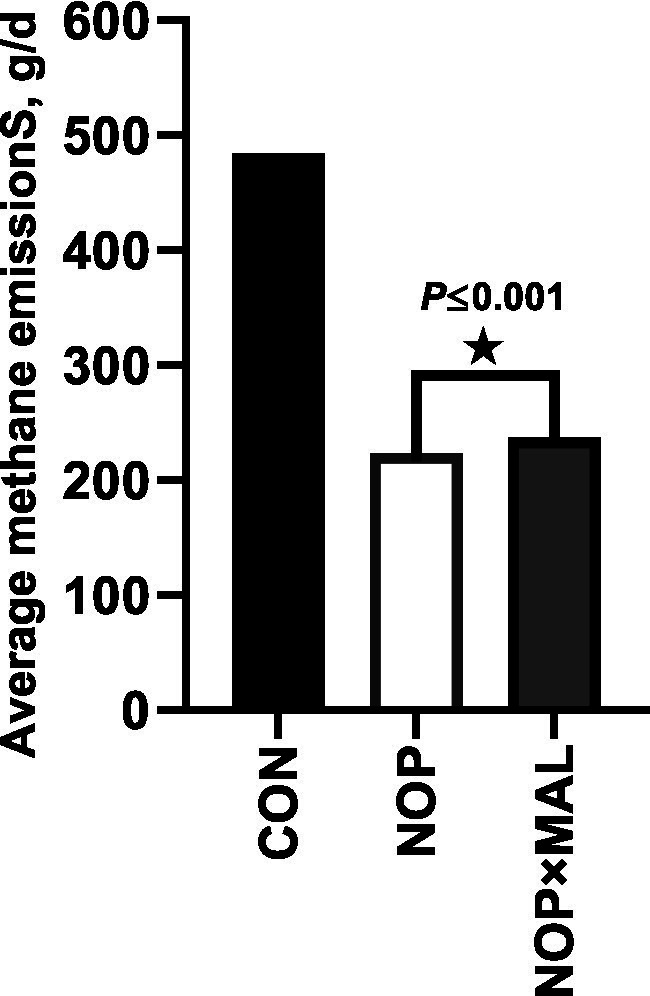
Methane emissions of three treatments in whole period.

In this experiment, cows were fed NOP (10% NOP 200 mg/kg feed dry matter) or NOP × MAL (10% NOP at 200 mg/kg feed dry matter plus 99% *L*-malate at 10 g/kg feed dry matter) via the TMR. Dairy cow DMI and milk yield were not affected by NOP (Table 2), which are results generally consistent with those of previous studies with NOP (19, 43, 44). In NOP × MAL, compared with CON, DMI decreased (*p* ≤ 0.001), but milk yield was not affected. Compared with CON, enteric CH_4_ emissions decreased by 54% in NOP and by 51% in NOP × MAL (Figure 1). Simultaneously, the H_2_ concentration in the two treatments also increased (*p* = 0.001). The continuous CH_4_ emission reduction effect of the two treatments is generally the same as that in previous studies with NOP (5, 14, 40). Treatment with NOP significantly increased milk fat concentration (*p* ≤ 0.001) compared with that in CON, but the increase in NOP × MAL was greater (*p* = 0.01) than that in NOP. In dairy cow serum, the activities of PK (*p* = 0.07) and CS (*p* = 0.07) increased slightly in NOP compared with that in CON, whereas the activities of MDH (*p* = 0.10), PCK (*p* = 0.13), PK (*p* = 0.05), and CS (*p* = 0.02) were higher or tended to be higher in NOP × MAL than in NOP. To explain the results, coenzyme M reductase (MCR) might be inactivated by the nitrooxy group in NOP, which would result in continuous reductions in CH_4_ emissions and simultaneous increases in the content of H_2_ (16). Hydrogen gas is considered a high-energy gas, and a small part of the H^+^ accumulated in the rumen can be converted into extra energy to increase milk fat concentration, which might be why milk fat concentration increased in NOP (35, 45). The compound *L*-malic acid is a hydrogen transporter, and as H_2_ accumulates in the rumen, hydrogen ions can be carried by the hydrogen transporter and transferred to mitochondria in rumen microbes and the mitochondria in the cow to be oxidized into ATP, which provides additional energy for animals to use in production. Therefore, in a previous study, the addition of malic acid to the diet of dairy cows not only reduced CH_4_ emissions but also increased milk production (46). Thus, the mechanism of malic acid might be explained. Similarly, activities of the enzymes MDH, PCK, PK, and CS in dairy cow blood serum were higher in NOP × MAL than in NOP. This result further indicated that NOP × MAL increased the processes of gluconeogenesis and glycolysis and activated the Krebs cycle and biological oxidation. As a result, NOP × MAL could provide more energy than NOP to increase milk fat concentration, which might explain why milk fat concentration in NOP × MAL was higher than that in NOP. In terms of CH_4_ emissions, both *in vivo* and *in vitro* experiments lead to the conclusion that malic acid can reduce CH_4_ emissions (29, 30). However, then reduction in CH_4_ emissions in NOP × MAL was slightly lower than that in NOP, which could be explained by a slight competitive antagonism between NOP and MAL in reducing CH_4_ emissions. In previous studies, malic acid increases the milk yield of dairy cows (27, 28, 47). However, contrary to the hypothesis and experimental conclusion in this study, NOP × MAL did not increase the milk yield of cows but instead reduced DMI and had no effect on milk yield. This result could be explained by the diet meeting the energy demand of dairy cows for production and not requiring further consumption (35).

**Table 2:** Effects of supplementing diets with 3-nitrooxypropanol (NOP) and 3-nitrooxypropanol plus *L*-malate (NOP × MAL) on dry matter intake (DMI), methane emissions, milk yield, body weight (BW), feed efficiency, milk composition, and serum enzyme activity of dairy cows.

	Treatment^1^				*p*-value^2^	
Item	CON	NOP	NOP × MAL	SEM	C vs. N	N vs. N × M
DMI, kg/d	24.5	23.6	22.5	0.27	0.10	0.05
Milk yield, kg/d	22.6	23.5	23.0	0.57	0.27	0.54
CH_4_, g/d	484.3	223.3	236.8	16.38	<0.001	0.84
CH_4_, g/kg of DMI	19.8	9.5	10.5	0.95	<0.001	0.58
H_2_, ppm/d	0.0	7.53	5.20	0.30	<0.001	0.001
ECM,^3^ kg/d	22.5	26.8	27.6	0.68	0.002	0.45
Milk NE_L_,^4^Mcal/d	16.7	20.0	20.6	0.51	0.002	0.44
ECM feed efficiency,^5^kg/kg	0.92	1.14	1.23	0.03	0.001	0.11
Feed efficiency,^6^kg/kg	0.92	1.00	1.02	0.02	0.057	0.45
Milk fat, %	3.75	4.74	5.09	0.06	<0.001	0.01
Milk protein, %	3.67	3.99	4.08	0.09	0.03	0.48
Milk lactose, %	4.74	4.72	4.85	0.03	0.88	0.10
MUN, mg/dL	21.3	19.8	19.4	0.31	0.02	0.67
BW, kg	660	655	652	2.70	0.43	0.67
The activity of enzyme,^7^						
MDH (U/L)	53.8	59.0	66.0	2.65	0.21	0.10
PCK (U/L)	112	120	130	3.06	0.24	0.13
PK (m U/L)	329	353	379	5.45	0.07	0.05
CS (U/L)	16.37	18.45	21.13	0.56	0.07	0.02

1 CON = Control.

2 C vs. N, CON vs. NOP; N vs. N × M, NOP vs. NOP×MAL.

3 ECM (kg/d) = kg of milk production × (383 × fat% + 242 × protein% + 165.4 × lactose% + 20.7) ÷ 3,140 (59).

4 Milk NE_L_ (Mcal/d) = kg of milk production × (0.0929 × fat% + 0.0563 × protein% + 0.0395 × lactose%) (60).

5 ECM yield ÷ dry matter intake (DMI).

6 Milk yield ÷ DMI.

7 MDH, malate dehydrogenase; PCK, phosphoenolpyruvate carboxykinase; PK, pyruvate kinase; CS, citrate synthase.

### 3.2 Effects of NOP and NOP × MAL to dairy cows on the volatile fatty acid (VFA) profile in rumen fluid and rumen microbial profile counts

Compared with CON, NOP tended to decrease (Table 3) the molar ratio of acetate (*p* = 0.08) and increase the molar ratio of propionate (*p* = 0.10). Simultaneously, compared with NOP, NOP × MAL had the same tendency to decrease the molar ratio of acetate (*p* = 0.07) and increase the molar ratio of propionate (*p* = 0.09). As a result, the molar ratio of acetate-to-propionate decreased in cows fed the two treatments. In addition, compared with CON, molar proportions of butyrate and valerate increased in NOP, which are results generally consistent with those of previous studies on NOP (18, 44). Simultaneously, compared with NOP, the molar ratios of butyrate and valerate tended to increase in NOP × MAL. Compared with CON, NOP reduced the concentration of N-NH_3_ (*p* = 0.02); however, there was no difference between NOP × MAL and NOP. Similarly, compared with CON, NOP tended to reduce copy numbers of total bacteria (*p* = 0.15) and methanogens (*p* = 0.13), but there was no distinction between NOP × MAL and NOP. This result could be explained by the fact that an increase of propionate in rumen fluid is considered to be a competitive alternative compared with an H_2_ sink (44, 48). The concentration of H_2_ in NOP × MAL and NOP was increased, but the H_2_ discharged from the rumen was only a small part of the H_2_ estimated by the two treatments to reduce CH_4_ emissions. Because of possible adaptation of rumen ecosystems, an increase in dissolved H_2_ concentration in the rumen is bound to replace H_2_ sinks and the incomplete recovery of reduction equivalent in discharged H_2_ (49, 50). Therefore, a decrease in the molar ratio of acetate and an increase in the molar ratio of propionate in this experiment were expected. In previous studies, malic acid decreased the molar ratio of acetate in rumen fluid and increased the molar ratio of propionate (29, 51). In this experiment, the acetate-to-propionate ratio in NOP × MAL was slightly lower than that in NOP, which might be explained by the synergistic effect of NOP and MAL in rumen fermentation. Melgar et al. (18) reported that valerate is produced by the condensation of acetate and propionate, which could explain why valerate increased in the two treatments. In addition, compared with NOP, the pH in NOP × MAL tended to increase, which indicated that malic acid increased the transport of hydrogen ions from H_2_ sinks in the rumen to cell mitochondria to be oxidized to ATP. However, there are also endosymbiotic and ecto-endosymbiotic relations between protozoa and some methanogens, indicating there are also relations between CH_4_ production and methanogens and protozoa (52). In studies on the effects of tea saponin and lipids, the diversity of methanogenic bacteria and CH_4_ production often decreased with reductions in protozoa (53–55). Although NOP did not affect the total numbers of protozoa, it decreased copy numbers of total methanogenic archaea and bacteria, indicating that NOP had a highly specific effect on total methanogenic archaea in this experiment. Note that although MAL was not the focus of discussion, the effect of NOP × MAL on dairy cows was worth explaining in this experiment.

**Table 3:** Effects of supplementing diets with 3-nitrooxypropanol (NOP) and 3-nitrooxypropanol plus *L*-malate (NOP × MAL) to dairy cows in the middle lactation period on the volatile fatty acid (VFA) profile in rumen fluid and rumen microbial profile counts.

	Treatment^1^				*p*-value^2^	
Item	CON	NOP	NOP × MAL	SEM	C vs. N	N vs. N × M
pH	5.48	5.40	5.63	0.05	0.43	0.11
Total VFA, mmol/L	108	107	105	1.20	0.71	0.52
VFA molar proportion (%)						
Acetate	65.80	62.70	59.30	1.13	0.08	0.07
Propionate	26.60	27.70	28.60	0.34	0.10	0.09
Butyrate	17.52	18.13	18.59	0.13	0.02	0.07
Isobutyrate	1.24	1.24	1.25	0.01	0.60	0.42
Valerate	2.28	2.34	2.38	0.01	0.04	0.10
Isovalerate	1.57	1.57	1.58	0.02	0.06	0.09
Acetate-to-propionate ratio	2.22	2.01	1.83	0.50	0.04	0.07
N-NH_3_, mg/100 mL	13.11	10.01	8.80	0.60	0.02	0.36
Total bacteria copy numbers, × 10^7^/g of rumen digesta	15.05	14.41	14.24	1.02	0.15	0.87
Methanogen copy numbers, ×10^6^/g of rumen digesta	1.82	1.60	1.67	1.01	0.13	0.79
Protozoa copy numbers, ×10^5^/g of rumen digesta	1.63	1.61	1.62	0.35	0.69	0.78

1 CON = Control.

2 C vs. N, CON vs. NOP; N vs. N × M, NOP vs. NOP×MAL.

However, in terms of N-NH_3_, compared with NOP (19, 44), NOP in this experiment reduced the concentration of N-NH_3_. This result might be because NOP improved the utilization efficiency of N-NH_3_ in dairy cows, which could also explain the increases in CP and decreases in MUN in milk.

### 3.3 Effects of NOP and NOP × MAL on fatty acids in milk of dairy cows (μg/mL of total fatty acids)

In terms of milk FAs (Table 4), NOP increased the short-chain FAs 4:0 and 10:0 (*p* = 0.001), 8:0 and 14:0 (*p* = 0.02), and the short-chain FAs 6:0 (*p* = 0.015) and 12:0 (*p* = 0.01). The NOP × MAL treatment resulted in a similar increase in milk FAs. Approximately 50% of the fat in milk comes from the absorption of FAs in the blood of dairy cows by mammary glands, with the other 50% from the *de novo* synthesis of FAs (56, 57). The main substrates for synthesizing FAs in dairy cows are acetate and butyrate, but butyrate only provides half of the four carbons (58). Therefore, the short-chain FAs in mammary glands are mainly synthesized from acetate. When CH_4_ production in the rumen is inhibited, butyrate seems to be the main substrate for synthesis of short-chain FAs in mammary glands (18), which is also the result of a decrease in CH_4_ emissions and an increase in the butyrate molar ratio. In addition, when CH_4_ production in the rumen was inhibited in NOP, biooxidation of a small part of the H_2_ sink might provide additional energy for the synthesis of FAs in milk. However, the NOP × MAL treatment could provide more additional energy for FAs synthesis, which might explain NOP × MAL had further the increase in FAs concentration in milk than NOP. In this experiment, the concentrations of monounsaturated fatty acids (MUFAs) 16:1 and 18:1 decreased in NOP, and thus, the concentration of total MUFAs also decreased. This result could be explained by the fact that biohydrogenation may provide a small absorption sink for H_2_ when CH_4_ production in the rumen is inhibited (43). Total concentrations of saturated fatty acids (SFAs), MUFAs, and polyunsaturated fatty acids (PUFAs) increased in NOP × MAL, which might be because when CH_4_ production in the rumen was inhibited, additional H_2_ sinks were transferred by MAL and eventually oxidized into extra energy to increase generation of SFAs, MUFAs, and PUFAs. In that situation, biohydrogenation seemed to be weakened compared with that in NOP. In addition, residues and metabolites of NOP in milk were not detected, which indicated that NOP can be completely metabolized by dairy cows and has no effect on animal health.

**Table 4:** Effects of supplementing diets with 3-nitrooxypropanol (NOP) and 3-nitrooxypropanol plus *L*-malate (NOP × MAL) on fatty acids in milk of dairy cows in the middle lactation period (μg/mL of total fatty acids).

	Treatment^1^				*p*-value^2^	
Item	CON	NOP	NOP × MAL	SEM	C vs. N	N vs. N × M
C4:0	44.0	60.1	84.8	1.90	0.001	<0.001
C6:0	31.8	40.3	76.5	1.96	0.015	<0.001
C8:0	26.5	32.6	48.6	1.40	0.02	<0.001
C10:0	45.4	56.5	78.9	1.40	0.001	<0.001
C12:0	67.6	73.9	86.2	1.11	0.01	<0.001
C14:0	225	267	398	10.25	0.02	<0.001
C14:1	25.36	22.48	32.1	0.92	0.13	<0.001
C15:0	31.1	28.3	40.1	1.97	0.59	0.01
C16:0	657	646	737	6.06	0.50	<0.001
C16:1	61.21	47.68	77.87	2.18	0.006	<0.001
C18:0	233	266	407	9.05	0.08	<0.001
C18:1	619	544	832	15.38	0.02	<0.001
C18:2	147	130	256	5.61	0.13	<0.001
C18:3n-3	33.0	35.9	39.4	0.79	0.07	0.04
C18:3n-6	22.0	23.8	35.5	1.12	0.52	<0.001
C20:0	29.0	31.2	40.8	0.48	0.03	<0.001
ΣSFAs^3^	1,440	1,553	2,048	17.29	0.004	<0.001
ΣMUFAs^4^	706	614	942	18.13	0.02	<0.001
ΣPUFAs^5^	202	190	331	5.62	0.30	<0.001

1 CON = Control.

2 C vs. N, CON vs. NOP; N vs. N × M, NOP vs. NOP×MAL.

3 ΣSFAs = Total saturated fatty acids.

4 ΣMUFAs = Total monounsaturated fatty acids.

5 ΣPUFAs = Total polyunsaturated fatty acids.

## 4 Conclusion

Supplementing with NOP (10% NOP 200 mg/kg feed dry matter) reduced CH_4_ in the guts of dairy cows by 54%, and supplementing with NOP × MAL (10% NOP at 200 mg/kg feed dry matter plus 99% *L*-malate at 10 g/kg feed dry matter) decreased CH_4_ in the guts of dairy cows by 51%. The NOP treatment did not affect DMI and milk yield, whereas the NOP × MAL treatment reduced DMI but did not affect milk yield. Both treatments reduced the ratio of acetate-to-propionate and tended to reduce copy numbers of methanogens, which could explain reductions in CH_4_ emissions. In addition, NOP increased the concentrations of short-chain FAs and total SFAs but decreased those of total MUFAs because of the action of a small part of rumen biohydrogenation. Compared with NOP, NOP × MAL increased the concentrations of short-chain FAs, total SFAs, MUFAs, and PUFAs, which indicated that NOP × MAL increased the energy utilization rate of cows compared with that with NOP.

## Data Availability

The original contributions presented in the study are included in the article/supplementary material, further inquiries can be directed to the corresponding authors.
